# Changes in the Vaccination Laws

**Published:** 1908-03-21

**Authors:** 


### Changes in the Vaccination Laws.

Four months ago we published a criticism of the new Vaccination Order, and a forecast of its probable effects. A Public Vaccinator in the South of England now draws our attention to this and other changes which the Local Government Board has made in the working of the Vaccination Act. He is surprised that so little public notice has been taken of a state of affairs which is likely to have important results in the near future. The leading facts are twofold. In the first place, the Local Government Board circulated during the early part of last year among the various Boards of Guardians a recommendation that after August 31, 1907, the fees paid to Public Vaccinators in respect of vaccinations should be materially reduced. The discretionary powers of the Guardians were not interfered with; but, as was only to be expected, this official suggestion coming from headquarters has been interpreted by the majority of the local authorities as little short of an order. The consequence is that, justly or unjustly, the Public Vaccinators throughout the greater part of the country are now receiving considerably reduced fees for their work. In the second place—and this is of far more importance, since it affects the safety of the whole community, and not merely the interests of one section of the medical profession—the President of the Local Government Board issued last year an Order, which came into force on January 1, 1908, modifying the administration of the Vaccination Act of 1898, in so far as it concerns exemption on the grounds of conscientious objection. Mr. Burns's new Order, roughly summarised, provides that as soon as a birth is registered the Vaccination Officer (who is nearly always also the local registrar of births and deaths) shall deliver to the parents a printed form, upon which either parent may declare his or her conscientious objection to the vaccination of the child as being prejudicial to its health. This declaration may be made by the father or the mother before a magistrate or a commissioner of oaths within four months of birth. It is not unreasonable to say of this Order that it offers every facility to parents to avoid the vaccination of their children. While under the original intention of the Act of 1898 ignorant and foolish parents were confronted with difficulties in the path of exemption, the Order of 1908 distinctly suggests to them that vaccination is an unnecessary burden, and encourages them to avoid it. What is the natural result of this? In many districts the number of conscientious objections has already materially increased; and the presumption is that, as the generous provisions of the Local Government Board become better known among the unintelligent classes, the diminution in the number of vaccinations will progress. The Vaccination Act of 1898 encouraged parents to have their children vaccinated, and inspired those responsible for carrying out the work to do their best. Experience showed that the Act was most efficient in its working. And now Mr. Burns's new Order has practically nullified it. If any further indictment of the present state of affairs were needed, we might add that the local Vaccination Officers, who, by the provisions of the Act, are rewarded for their work by capitation fees for successful vaccinations, are now given a great deal of extra work under the new Order, while, owing to the diminished numbers of vaccinations, their remuneration is less than it was. It is certainly high time that public attention was drawn to these things. When the next epidemic of small-pox comes it may be too late. By that time the bulk of the child population of the country may be unprotected. If this happens Great Britain will be the laughing stock of other and less sentimental Governments. Our Ministers may then recognise, when the harm has been done, that it is cruel to flatter the lower classes by leaving to them the decision in matters which concern the public health and safety. The average working man or woman is entirely incapable of forming a valid conscientious objection to vaccination or to any other hygienic procedure.

